# p75 Neurotrophin Receptor in the Skin: Beyond Its Neurotrophic Function

**DOI:** 10.3389/fmed.2017.00022

**Published:** 2017-03-07

**Authors:** Carlo Pincelli

**Affiliations:** ^1^Laboratory of Cutaneous Biology, Department of Surgical, Medical, Dental and Morphological Sciences, University of Modena and Reggio Emilia, Modena, Italy

**Keywords:** p75 neurotrophin receptor, CD271, neurotrophins, skin, epidermis, homeostasis, melanoma, psoriasis

## Abstract

p75 neurotrophin receptor (p75^NTR^), also known as CD271, is the low-affinity receptor that, together with the tyrosine kinase receptor tropomyosin-receptor kinase (Trk), mediate neurotrophin (NT) functions. Beside their classic role in skin innervation, NT and their receptors constitute a complex cutaneous network associated with a number of autocrine and paracrine activities. In this context, the role of p75^NTR^ is becoming more and more important. This review will focus on the intriguing functions of p75^NTR^ in healthy and diseased skin. First, p75^NTR^ counterbalances the proliferative and survival activities of its cognate receptor Trk by inducing keratinocyte apoptosis. In addition, p75^NTR^ identifies an early transit-amplifying (TA) keratinocyte population and plays a critical role in keratinocyte stem cell transition to its progeny as well as in epidermal differentiation. p75^NTR^ is absent in psoriatic TA cells, thus rendering these cells resistant to apoptosis. On the other hand, p75^NTR^ infection restores NT-induced apoptosis in psoriatic keratinocytes. Taken together, these results provide evidence for a critical role of p75^NTR^ in epidermal homeostasis, while its lack may account for the TA defect in psoriasis. While the issue of p75^NTR^ as a marker of melanoma initiating cells is still to be solved, there is strong evidence that downregulation of this receptor is a precondition to melanoma invasion and metastasis *in vitro* and *in vivo*. All in all, this review points to p75^NTR^ as a major actor in both physiologic and pathologic conditions at the skin level.

## Introduction

The neurotrophin (NT) family of growth factors that includes nerve growth factor (NGF), brain-derived neurotrophic factor (BDNF), NT-3, and NT-4, plays a fundamental role in the development and maintenance of the nervous system (1). Each NT exerts its activities through two receptor classes: the high-affinity tropomyosin-receptor kinase (Trk) and the common p75 NT receptor (p75^NTR^, also known as CD271) (2).

p75^NTR^ belongs to the tumor necrosis factor receptor family and interacts with a variety of ligands and co-receptors to mediate a range of functions, although this interplay is complex and still poorly understood (3). After ligand activation, p75^NTR^ is proteolytically cleaved by γ-secretase to give intracellular domain (ICD) that is responsible for specific signaling (4). Heterodimerization of p75^NTR^ with Trk increases the NT/Trk interaction affinity, thus augmenting growth and survival functions. In addition, pro-NTs bind to the sortilin-p75^NTR^ complex and initiate cell death signaling (5). ICD in itself can operate independently of the other co-receptors and the functional activity of p75^NTR^ depends on its subcellular localization, on the final location of the fragment (6, 7), and on which partners it is associated (8). p75^NTR^ interacts with a variety of proteins (9) that in turn determine signaling through different pathways (10–12). This interplay allows p75^NTR^ to play a flexible, but pivotal role in the regulation of multiple activities and ultimately the fate of the cell. The present review will focus on the current knowledge on p75^NTR^ in healthy and diseased skin.

## p75^NTR^ Mediates NT-Sustained Skin Innervation

In the peripheral nervous system, the survival of sensory and sympathetic neurons largely depends on the production by innervated target of NGF and its cognate NTs (13). Overexpression of NGF in skin determines increase of sensory innervation (14). NGF production is proportional to the innervation density, and it is retrogradely transported to the cell body of the neuron where it regulates its maintenance (15). Also in skin, Trk receptors mediate NTs-enhanced cell survival, while p75^NTR^ promotes cell death of sensory and sympathetic neurons (16). Mice carrying a mutation of gene encoding p75^NTR^ display a marked decrease in sensory cutaneous innervation, associated with the development of ulcers in the distal extremities (17), indicating a critical role of p75^NTR^ in the survival and functions of sensory neurons. In human skin, the intensity of p75^NTR^ immunoreactivity in sensory nerves is stronger in areas where NGF is upregulated in target cells (18). Moreover, p75^NTR^ is strongly increased in sensory fibers in conditions where keratinocytes express high levels of NGF (19).

## p75^NTR^ Outside the Nervous System

Beside the classical role of maintaining neuronal cells, NT and their receptors possess a range of functions outside the nervous system. Most non-neuronal cells express NT receptors and respond to NT stimuli, which implies for these neural substances, the role of actual growth factors and/or mediators in a number of physiologic (20) and pathologic conditions (21, 22). On the other hand, the function of p75^NTR^ signaling alone or in combination with its co-receptors outside the nervous system has been largely overlooked and remains to be clarified.

## NT Network in the Skin

Over the past 20 years, it has become clear that virtually every cutaneous cell synthesizes and releases NTs and expresses their receptors. Indeed, NGF is produced in basal keratinocytes and is involved in important autocrine functions (23, 24). Also, the other NTs are detected in keratinocytes where they exert similar activities (25).

In addition, the important observation that human melanocytes express all NTs and their receptors (26, 27) has been confirmed by the critical role these molecules and their receptors play in melanogenesis (28) and melanin production (27). This is further supported by the demonstration that NGF rescue melanocytes from apoptosis (29) and stimulates their migration and dendricity (30). Recently, Byun and co-workers have shown that NGF increases melanogenesis and plays a role in the pathogenesis of melasma (31). Interestingly, NTs and in particular p75^NTR^ are expressed in neural crest (NC) cells, the melanocyte precursors (32).

While the role of NGF in wound repair has been known for a long time (33), NTs stimulate fibroblasts (34), one of the most important cell involved in this process. Myofibroblasts produce all NT and their receptors. Both p75^NTR^ and Trks mediate fibroblast proliferation, differentiation, and migration. In addition, NGF or BDNF increase the tensile strength in a collagen gel (35), while tensile stimuli increase NGF in human fibroblasts (36). Recently, p75^NTR^ has been shown to co-immunoprecipitate with the pro-inflammatory phosphodiesterases-4 in myofibroblasts (37), although the activities of this complex remain to be clarified.

Aloe and Levi-Montalcini originally observed that NGF enhances the number of mast cells in tissues (38). Since then, it is well accepted that there is a close contact between nerves and mast cells to form the “mast cell-nerve unit” that seems to play a key role in physiologic and pathophysiologic processes (39), with particular regard to itch and atopic dermatitis (40). Indeed, high levels of NT-3 are expressed in atopic dermatitis mast cells (41), and p75^NTR^ is induced in lesional atopic mast cells (42).

These findings strongly indicate the presence of a complex NT network in the skin responsible for a number of autocrine and paracrine functions. In this context, a role for p75^NTR^ has recently begun to emerge.

## p75 and Hair Follicle

Neurotrophins and their receptors are involved in hair follicle morphogenesis in a complex manner. Consistent with the opposite roles of the two NT receptors, it would appear that NGF/TrkA promotes an anagen supporting role, whereas proNGF/p75 interaction is associated with a catagen-promoting effect (43). Moreover, NGF, but not BDNF, accelerates hair follicle development (44). On the other hand, BDNF inhibits hair shaft elongation and provokes catagen (45). NGF expression is also observed in keratinocytes of human hair follicles (46), with important implications for morphogenesis (47). Adly and co-workers reported that p75^NTR^ protein displays a hair cycle-dependent fluctuation in human scalp (48), and p75^NTR^ induces hair follicle involution *via* apoptosis (49).

## p75^NTR^ and Epidermal Homeostasis

Epidermal homeostasis is based on a fine balance between keratinocyte proliferation, differentiation, and apoptosis (50). Constant epidermal regeneration is achieved by stem cells that are slow-cycling and possess the capacity of self-renew (51). Keratinocyte stem cells (KSC) reside in the basal layer and generate transit-amplifying (TA) cells that undergo a limited number of cell divisions before committing to terminal differentiation (52), although this model has been recently questioned (53, 54).

Psoriasis is an immune-mediated dermatosis where alterations of epidermal homeostasis account for the main pathologic and clinical outcome. Indeed, psoriasis is characterized by keratinocyte hyperproliferation, abnormal differentiation, and increased resistance to apoptosis (55), resulting in excessive epidermal thickness, the key feature of psoriatic plaque. Thus, psoriasis is a perfect model of altered epidermal homeostasis that can be exploited for evaluating the expression and function of several molecules, including NTs.

Normal human keratinocytes synthesize and secrete all NTs and express their receptors. NGF is predominantly expressed in KSC (25), while TrkA is located only in basal keratinocytes with a uniform pattern (56). On the other hand, p75^NTR^ is expressed in a subpopulation of basal keratinocytes with an irregular distribution (57). NGF levels are increased in psoriatic tissue (58) and keratinocytes (59). TrkA receptor is overexpressed throughout the epidermal layers in psoriatic skin (56), whereas p75^NTR^ expression completely disappears in lesional psoriatic epidermis (57). These findings might be consistent with the general concept on the opposing effects Trks and p75^NTR^ mediate in the nervous system (60). The following data confirm that this is actually the case also at the skin level.

Indeed, NGF or NT-3 stimulates keratinocyte growth (25, 61), and transfecting HaCat cells with TrkA enhance cell proliferation (62), indicating that NTs act as mitogens through their high-affinity receptor. Consistently, K252, a natural alkaloid that blocks Trk phosphorylation, thus inhibiting NT functions, prevents NGF-induced keratinocyte proliferation (23). In line with the increased expression of Trk and NGF (63) in psoriasis, topical treatment with K252 improves psoriasis in the immunodeficient mouse–human skin model (64). In psoriasis, keratinocyte apoptosis is spontaneously decreased (65), and psoriatic keratinocytes are resistant to apoptosis (66). Recently, PageRank analysis revealed a group of hub genes with anti-apoptotic functions in psoriasis (67).

**Figure 1:**
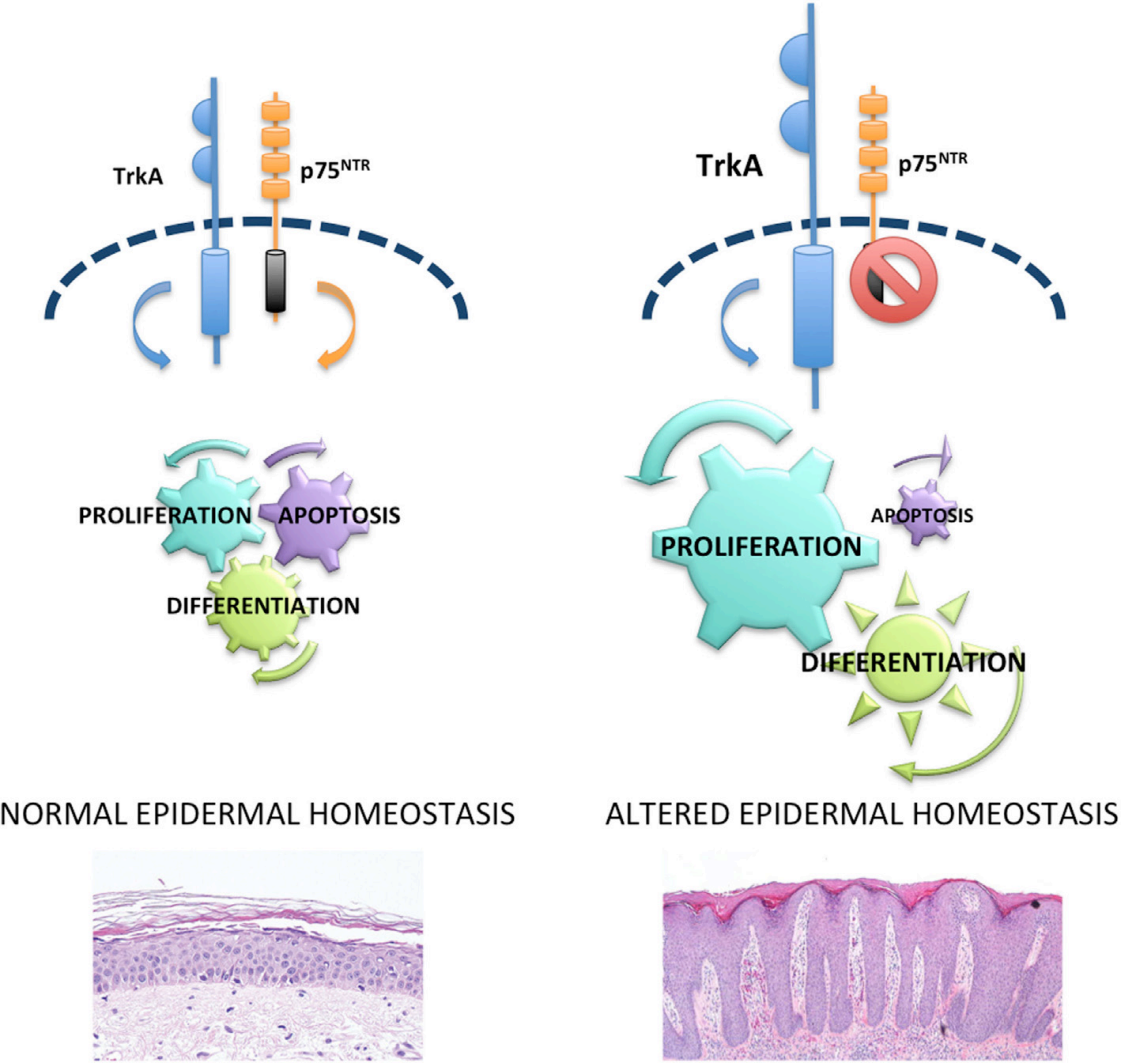
**p75^NTR^ in epidermal homeostasis and psoriasis**. In normal epidermis, there is a balance between p75^NTR^ and tropomyosin-receptor kinase (Trk) receptors that controls proliferation, differentiation, and apoptosis and allows a perfect homeostasis. Upregulation of Trk and lack of p75^NTR^ cause hyperproliferation, abnormal differentiation and reduced apoptosis that in turn are responsible for the altered epidermal homeostasis typical of psoriasis.

Endogenous NGF acts as a survival factor for human keratinocytes through Trk receptor, as K252 induces cell death in these cells, by maintaining constant levels of the anti-apoptotic protein Bcl-2 (24). Furthermore, NGF protects keratinocytes from ultraviolet-B-induced apoptosis by preventing the cleavage of the enzyme poly (ADP-ribose) polymerase (61). Taken together, these data support the notion that NTs and Trk receptors mediate proliferative and survival activities in human keratinocytes. Abnormal mitotic and apoptotic processes mediated by NTs lead to the imbalanced epidermal homeostasis resulting in the excessive epidermal thickening observed in psoriasis (Figure 1).

The absence of p75^NTR^ as opposed to the increased Trk expression in lesional psoriatic epidermis (57) supports the hypothesis that an imbalance of the NT receptors could play an important role in the alterations of epidermal homeostasis observed in psoriasis. In normal human skin, binding of p75^NTR^ with proper ligands mediates keratinocyte apoptosis. In fact, β amyloid, able to bind directly to p75^NTR^ (68), activates caspase-3 only in keratinocytes expressing the low-affinity NT receptor. In addition, BDNF or NT-4, which signals only through p75^NTR^, induces apoptosis in human keratinocytes (57). In the nervous system, when both NT receptors are expressed, NT binds to Trk/p75 complex and mediate survival (69). On the other hand, in normal human keratinocytes, where both receptors are expressed, it appears that p75^NTR^ can signal independently of Trk. This is in line with other studies in non-neuronal epithelial tissues. In fact, BDNF was recently shown to induce apoptosis in gingival epithelium *via* p75^NTR^ (70), and p75^NTR^ mediates apoptosis in prostate cancer cells (71), in the presence of Trk receptors. p75^NTR^ independent signal mediating apoptosis in keratinocytes is further confirmed by the co-immunoprecipitation with its interacting protein NRAGE (57) that is known to disrupt p75^NTR^-Trk complex and to cause cell death through JNK-dependent pathway (72). In keeping with this concept, BDNF induces apoptosis in human keratinocytes through the phosphorylation of JNK (57). Taken together, these results indicate that p75^NTR^ counteracts the survival and proliferative activities of Trk receptors, thus contributing to a normal epidermal homeostasis (Figure 1).

In human epidermis, differentiation begins when the transition from KSC to TA cells occurs, although the mechanisms underlying this process are still partially unknown.

p75^NTR^ is predominantly expressed in the keratinocyte subpopulation enriched in TA cells (57). p75^NTR^-positive keratinocytes sorted from freshly isolated TA cells still retain KSC markers, such as survivin and keratin 15, while they express less markers of differentiation, as compared to p75^NTR^-negative cells. In addition, p75^NTR^-positive TA cells display a higher proliferative capacity and a better colony forming efficiency, as compared to p75^NTR^-negative cells. Finally, human reconstructed epidermis derived from p75^NTR^-positive TA cells express markers of early differentiation (73). This indicates that p75^NTR^ identifies a population of early TA cells. A population of early TA has been already detected in the hair follicle (74) and in the interfollicular epidermis (75) and appears to be critical in the first steps of the differentiation process (76). p75^NTR^ protein that exerts its activities at the boundary between KSC and TA could function as early trigger of keratinocyte differentiation. In fact, silencing p75^NTR^ prevents calcium-induced keratinocyte differentiation and converts TA cells into a KSC phenotype. Moreover, overexpression of p75^NTR^ in KSC results in a keratinocyte subpopulation with the features of TA cells (73). These results indicate that p75^NTR^ could act as a “switch on-off” protein that critically regulates KSC-progeny transition and differentiation in human epidermis (Figure 1).

Excessive expansion of the TA cells compartment has been recently described in psoriatic skin (77), where a defect in TA subpopulation seems to account for the epidermal abnormalities observed in the disease (78). In addition, *in silico* studies have simulated psoriasis by altering the TA cells (79), and psoriatic TA cells are more advanced in their life cycle than their normal counterpart (80). p75^NTR^ levels are strikingly reduced in psoriatic TA (57), and the lack of p75^NTR^ seems to account for the reduced apoptosis of psoriatic keratinocytes. Indeed, BDNF fail to induce cell death in these cells, while overexpression of p75^NTR^ restores their susceptibility to apoptosis (73). These findings suggest that alterations of the TA cell in psoriasis are at least in part due to a defect in p75^NTR^. Interestingly, skin equivalent models derived from p75^NTR^-negative TA cells display a psoriasiform phenotype (73) in line with the absence of the receptor protein in psoriasis (57). Because p75^NTR^ plays a critical role in the early keratinocyte differentiation, it is tempting to speculate that the intrinsic defects in psoriatic epidermis occur in the early TA cells where the absence of p75^NTR^ may account for the altered epidermal homeostasis of the disease. It has been shown that when keratinocytes exit the niche, they could undergo either differentiation or programmed cell death (81). Although p75^NTR^ is clearly involved in both processes in human epidermis, the different triggers and pathways associated with the functions of the receptor remain to be elucidated.

## p75^NTR^ and Melanoma: More than Just a Marker

Melanoma cell lines synthesize and secrete all NTs and express NT receptors (82). p75^NTR^ was first isolated from a melanoma cell line, and it has become a useful tool for immunohistochemical diagnosis of melanoma (83). High degree of p75^NTR^ expression allows a better diagnosis of desmoplastic melanoma (84) and the distinction between spindle melanoma and other spindle cell tumors (85).

According to the “cancer stem cell” theory, a distinct subpopulation of melanoma cells (melanoma initiating cells, MIC) would account for the high tumorigenic properties, tumor heterogeneity, invasiveness, and drug resistance (86). p75^NTR^ has received a special attention as a possible MIC marker because it is a marker of NC cells, the melanocyte precursors (87), and for the similarities between NC stem cells and melanoma cells (88). Yet, whether p75^NTR^ identifies a group of highly tumorigenic MIC has not been clarified. It was first demonstrated that MIC express high levels of p75^NTR^ and p75^NTR^-positive, but not p75^NTR^-negative transplanted melanoma cells are capable of inducing metastasis *in vivo* (89). On the other hand, Quintana and co-workers could not confirm these data, by showing that p75^NTR^ -positive or p75^NTR^ -negative melanoma cells have the same tumorigenic potential (90). Recently, Boyle and colleagues, using different patient-derived xenograft assays, have clearly shown that p75^NTR^-negative and p75^NTR^-positive melanoma cells from each of the patients had similar tumorigenic activity, concluding that p75^NTR^ expression is unstable and not associated with increased tumorigenicity (91). This work also questions its role as a marker of melanoma aggressiveness (91). Consistently, p75^NTR^ expression inversely correlates with hypoxia and melanoma invasiveness *in vivo* (92).

**Figure 2:**
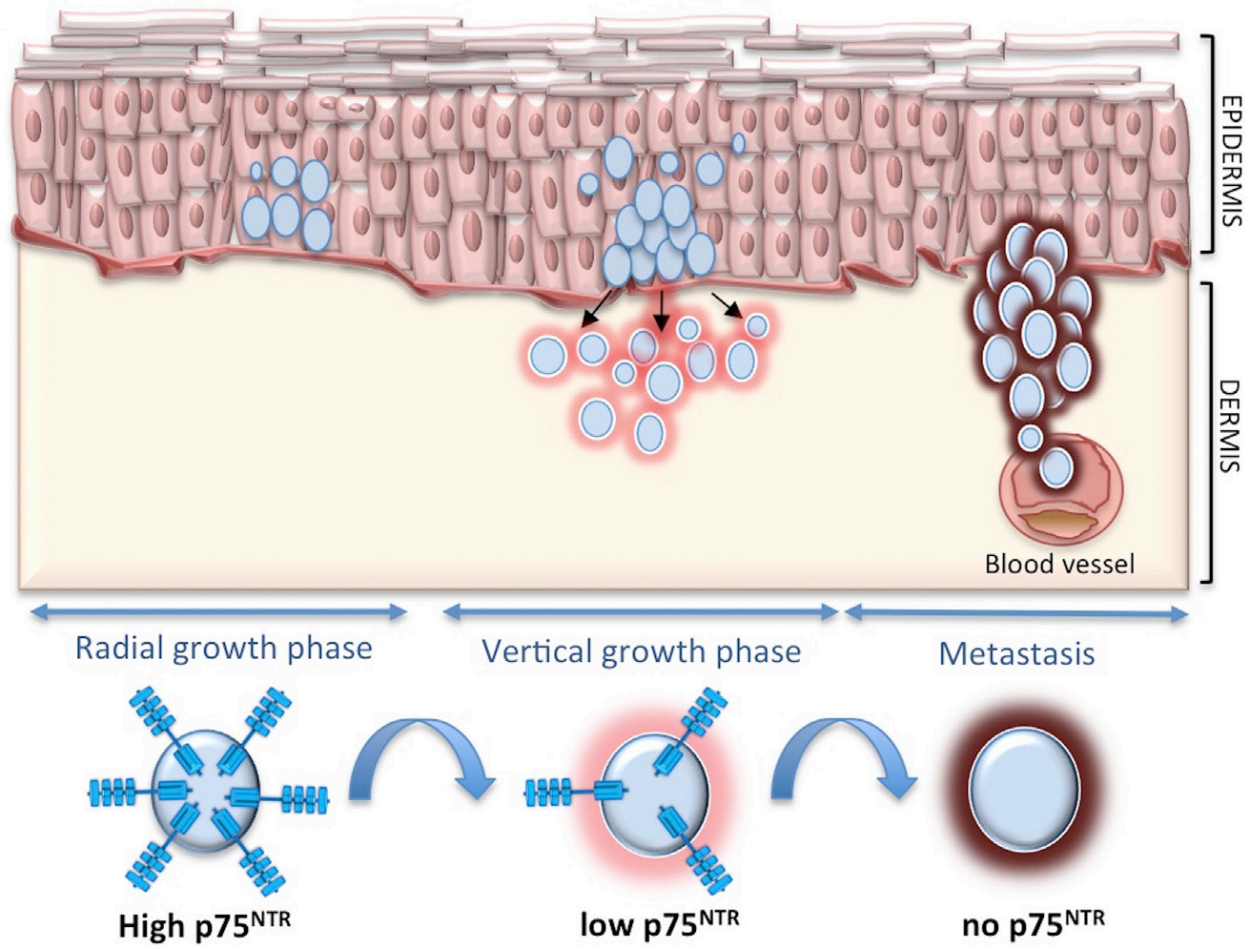
**p75 neurotrophin receptor (p75^NTR^) and melanoma**. p75^NTR^ is associated with less aggressive melanoma, while it tends to decrease and disappear during melanoma invasion and metastasis.

To definitely assess the role of p75^NTR^ in melanoma, we have recently carried out an extensive study *in vitro* and *in vivo* (93). In skin equivalent models, p75^NTR^ is highly expressed in early melanomas at the epidermal level and tends to disappear when melanoma starts to invade the dermis. In addition, p75^NTR^ is completely absent in skin reconstructs derived from metastatic cell lines. p75^NTR^ expression is highest in spheroids derived from primary melanoma cells, it decreases in cells derived from metastatic melanomas to disappear in highly invasive spheroids. p75^NTR^-negative cells show greater proliferation and invasiveness *in vitro* and are associated with a higher number of metastases in zebrafish, as compared with p75^NTR^-positive cells. Moreover, silencing p75^NTR^ induces a more aggressive phenotype in spheroids and in the animal model. By contrast, p75^NTR^ overexpression reduces invasiveness *in vitro* and strikingly reduces the number of metastases in zebrafish. This seems to indicate that p75^NTR^ switch off is critical for melanoma progression and metastasis (Figure 2).

## Conclusion

There is compelling evidence that, in the context of the skin NT network, p75^NTR^ is a major actor in both physiological and pathological conditions. In healthy skin, p75^NTR^-triggered differentiation and de-differentiation during KSC to progeny transition awaits confirmation in other epidermal stem cell compartments as well as in the mouse model. In addition, more studies are needed to understand the mechanisms underlying the absence of p75^NTR^ protein in psoriasis, and whether the lack of the receptor, that is critical for epidermal homeostasis, is associated with a psoriatic phenotype also *in vivo*.

While the downregulation of p75^NTR^ as a precondition for melanoma progression and metastasis is unquestionable, the molecular mechanisms associated with this function are not fully clarified. The low levels of β_1_ integrin and the decreased of cell-to-cell adhesion in the absence of p75^NTR^ could predispose melanoma to increased invasiveness (93). Furthermore, melanoma is characterized by an alteration of the apoptotic machinery. The lack of p75^NTR^ that exerts pro-apoptotic functions in melanoma (94), could favor tumor cell survival and metastasis.

## Author Contributions

The author confirms being the sole contributor of this work and approved it for publication.

## Conflict of Interest Statement

The author declares that the research was conducted in the absence of any commercial or financial relationships that could be construed as a potential conflict of interest.
